# Clinical Effectiveness, Clinical Stability, and Effects on Serum Galectin-7 Levels of Dupilumab and JAK Inhibitors in Moderate-to-Severe Atopic Dermatitis: A Real-World, Single-Center Analysis

**DOI:** 10.3390/medicina61050926

**Published:** 2025-05-20

**Authors:** Akihiro Horie, Tomomitsu Miyagaki, Chikako Hiranuma, Mami Iijima, Yoshiaki Hara, Shinya Oba, Mina Hashimoto, Reina Omori, Tatsuro Okano, Takafumi Kadono

**Affiliations:** Department of Dermatology, St. Marianna University School of Medicine, 2-16-1 Sugao, Miyamae-ku, Kawasaki 216-0015, Japan

**Keywords:** atopic dermatitis, dupilumab, JAK inhibitor, baricitinib, upadacitinib, POEM, DLQI, skin barrier dysfunction, galectin-7, stability

## Abstract

*Background and Objectives:* Several biologics and oral Janus kinase (JAK) inhibitors have been developed and shown in clinical trials and real-world studies to be effective and safe in moderate-to-severe atopic dermatitis (AD). In this study, we aimed to evaluate the real-world outcomes of patients with moderate-to-severe AD treated with dupilumab and JAK inhibitors in our facility, focusing on their short-term effect on serum galectin-7 levels, a biomarker reflecting skin barrier impairment, and one-year stability based on patient-oriented outcomes. *Materials and Methods:* In a single-center, retrospective study of AD patients treated with dupilumab or JAK inhibitors between January 2018 and December 2024, we assessed physician-oriented outcomes until 16 weeks and patient-oriented outcomes until 52 weeks. Serum galectin-7 levels at baseline and 4 and/or 16 weeks after treatment were measured in 14 patients. *Results:* A total of 45 patients starting dupilumab and 10 patients starting JAK inhibitors were enrolled. Percentage reductions in EASI scores from baseline at 4, 8, and 16 weeks were 58.36 ± 22.09, 69.59 ± 20.96, and 75.98 ± 19.70, with no significant differences between patients treated with dupilumab and JAK inhibitors. Serum galectin-7 levels were significantly reduced after treatment at 4 and 16 weeks in the entire population. Both DLQI and POEM scores were reduced at 4 weeks and gradually decreased until 52 weeks. The reduction was faster with JAK inhibitors than with dupilumab. Visits with unstable effectiveness, defined as a visit with a three-point or greater increase in the POEM score at 28, 40, and 52 weeks, were more frequent in JAK inhibitor patients. *Conclusions:* Both dupilumab and JAK inhibitors showed high effectiveness on skin inflammation and decreased a marker of skin barrier dysfunction within 16 weeks. JAK inhibitors improved patient-reported outcomes more quickly than dupilumab, but instability of effectiveness during 16 and 52 weeks was higher with JAK inhibitors.

## 1. Introduction

Atopic dermatitis (AD) is a common inflammatory skin condition, characterized by skin dryness, recurrent eczematous lesions, and severe pruritus, and its prevalence is increasing worldwide [1,2]. AD typically follows a pattern of flare-ups and remissions, with symptoms varying in severity over time, and has a significant impact on patients’ quality of life (QoL). The pathophysiology of AD is complex and multifactorial, and an interplay between skin barrier dysfunction, occasionally caused by loss-of-function variants of the filaggrin gene, and an enhanced type 2 immune response, represented by an increase in IL-4, IL-13, IL-31, IL-33, and thymic stromal lymphopoietin, plays a central role in the development of the disease [1,2,3]. Focusing on the suppression of type 2 immune responses, several biologics targeting IL-4 and/or IL-13 and oral Janus kinase (JAK) inhibitors have been developed and shown in clinical trials to be effective and safe in moderate-to-severe AD [4,5,6,7,8,9]. The approval of these biologics, such as dupilumab, tralokinumab, and lebrikizumab, alongside oral JAK inhibitors like baricitinib, upadacitinib, and abrocitinib, has provided clinicians with a wider range of targeted treatment options. There have been several reports of real-world data showing the effectiveness on skin inflammation and pruritus of these drugs in clinical settings and, interestingly, some have demonstrated even better results than those seen in clinical trials [10,11,12,13,14]. However, the effect on skin barrier dysfunction and the stability of effectiveness of these drugs in real-world settings remain areas of ongoing investigation. This single-center retrospective analysis aims to evaluate the real-world outcomes of patients with moderate-to-severe AD treated with dupilumab and JAK inhibitors, with a specific focus on their short-term effect on serum galectin-7 levels, a potential biomarker reflecting IL-4/IL-13–induced skin barrier impairment [15], and one-year stability of effectiveness based on patient-oriented outcomes during the first year.

## 2. Methods

### 2.1. Study Design and Patients

This single-center retrospective study incorporated serum samples and data from the medical records of patients with moderate-to-severe AD for whom treatment with dupilumab or JAK inhibitors including baricitinib and upadacitinib was initiated in our outpatient clinic specializing in AD from January 2018 to December 2024. Additional eligibility criteria for enrolment were a total eczema area and severity index (EASI) score of greater than 16, an Investigator’s Global Assessment (IGA) score of 3 or greater, and an affected body surface area (BSA) of greater than 10%. Patients who switched or discontinued these systemic therapies within 16 weeks and patients with a history of their previous use were excluded. In patients who switched or discontinued these systemic therapies, or who dropped out of our follow-up after 16 weeks of treatment, the data prior to these events were included in the analyses. Patients who started dupilumab received one 600 mg loading dose, followed by 300 mg of dupilumab subcutaneously every two weeks. Baricitinib was given orally at a dose of 4 mg/day. Upadacitinib was administered orally at a dose of 15 mg/day, which was increased to 30 mg/day in some patients. Data extracted from medical records included age at starting dupilumab or JAK inhibitors (years), sex, IGA score at baseline, %BSA at baseline, EASI score at baseline and 4, 8, and 16 weeks after treatment, patient-oriented eczema measure (POEM) score at baseline and 4, 8, 16, 28, 40, and 52 weeks after treatment, Dermatology Life Quality Index (DLQI) score at baseline and 4, 16, 28, 40, and 52 weeks after treatment, and laboratory data at baseline including disease severity markers of AD, such as serum lactate dehydrogenase (LDH), IgE, thymus and activation-regulated chemokine (TARC) levels, and eosinophil counts in peripheral blood. These markers were measured in the laboratory of our institution as part of routine medical practice. Specifically, serum LDH levels were measured by the International Federation of Clinical Chemistry method; serum IgE levels were measured by a fluorescence enzyme immunoassay; serum TARC levels were measured by a chemiluminescence enzyme immunoassay; and eosinophil counts in the peripheral blood were measured by flow cytometry using volume, conductivity, and scatter technology. The proportion of patients who achieved at least a 50%, 75%, or 90% reduction in the EASI score at 4, 8, and 16 weeks after treatment from baseline (EASI 50, EASI 75, or EASI 90, respectively) was calculated. A visit with unstable effectiveness was defined as a visit with a three-point or greater increase in the POEM score at 28, 40, and 52 weeks after treatment compared to the POEM score at 16, 28, and 40 weeks after treatment, respectively. Informed consent was obtained in the form of opt-out. Serum samples were collected before the start of systemic therapies and at 4 and 16 weeks of therapies in some patients after obtaining written informed consent from each patient. The medical ethical committee of St. Marianna University School of Medicine approved all described studies (5970 and 6746), and the study was conducted according to the principles of the Declaration of Helsinki.

### 2.2. ELISA

Galectin-7 in serum was quantified using a Human Galectin-7 ELISA Kit (Invitrogen, Waltham, MA, USA). These assays employ the quantitative sandwich enzyme immunoassay technique. Seum galectin-7 levels below the lower detection limit (57.9 pg/mL) were considered as 0 pg/mL.

### 2.3. Statistical Analysis

Differences between the two groups were analyzed using the Mann–Whitney U-test for quantitative variables and Fisher’s exact test for categorical variables. Differences before and after treatment were analyzed using the Wilcoxon signed-rank sum test. Correlation coefficients were determined using the Spearman’s rank correlation test. Statistical significance was set at *p* < 0.05.

## 3. Results

### 3.1. Patient Characteristics

Fifty-five patients were enrolled in this study. The patients’ backgrounds are shown in Table 1. The mean age of the patients was 32.2 ± 15.6 years, and 41 patients (74.5%) were male. EASI scores, DLQI scores, and POEM scores at baseline were 25.6 ± 7.03, 12.28 ± 7.03, and 17.74 ± 6.99, respectively. None of the patients had previously received biologics or JAK inhibitors. A total of 45 patients started dupilumab, and 10 started JAK inhibitors. Among JAK inhibitors, baricitinib was given to five patients and upadacitinib to five patients. All patients used concomitant topical therapies including emollient, steroid, tacrolimus, delgocitinib, and difamilast. No significant differences in age, sex distribution, EASI scores, DLQI scores, and POEM scores were found between the two groups. All patients continued these systemic therapies at 16 weeks, and 37 at 52 weeks. Seven patients were still on treatment but not for 52 weeks at the end of the observation period. Between 16 and 52 weeks, 11 patients stopped or changed systemic therapies, which was due to an inadequate response in seven patients, patient preference in two patients, and a significant response in two patients.

### 3.2. Short-Term Effectiveness of Dupilumab and JAK Inhibitors Based on the EASI Score

**Figure 1:**
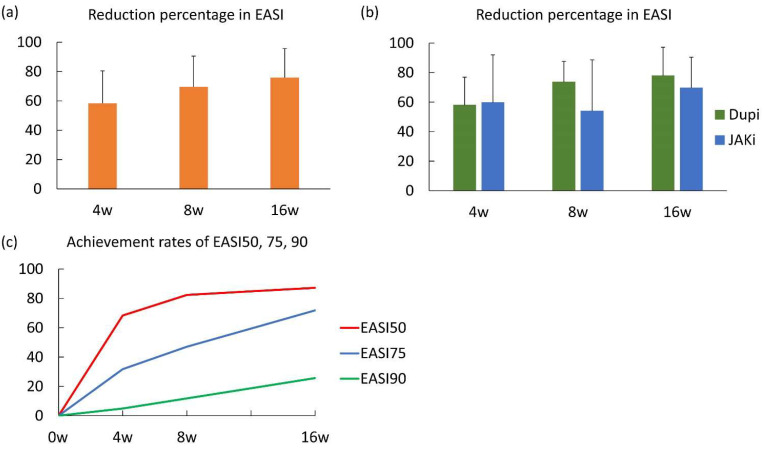
Short-term effectiveness of dupilumab and JAK inhibitors based on the eczema area and severity index (EASI) score. (**a**) Percentage reductions in the EASI score from baseline at 4, 8, and 16 weeks in the entire population. Data are presented as mean ± standard deviation. (**b**) Percentage reductions in the EASI score from baseline at 4, 8, and 16 weeks in patients treated with dupilumab and JAK inhibitors. Data are presented as mean ± standard deviation. (**c**) The achievement rates of EASI50, EASI75, and EASI90 at 4, 8, and 16 weeks in the entire population.

The percentage reductions in the EASI score from baseline for all patients enrolled at 4, 8, and 16 weeks were 58.36 ± 22.09, 69.59 ± 20.96, and 75.98 ± 19.70 (Figure 1a). In patients treated with dupilumab compared to those with JAK inhibitors, the percentage reductions in the EASI score at 4, 8, and 16 weeks were 57.99 ± 18.96 vs. 59.69 ± 32.33, 73.63 ± 14.05 vs. 53.98 ± 34.69, and 77.88 ± 19.30 vs. 69.63 ± 20.82, respectively (Figure 1b), and the trend was statistically similar in both groups. The achievement rates of EASI50 in the entire population at 4, 8, and 16 weeks were 68.29%, 82.35%, and 87.18%; those of EASI75 were 31.70%, 47.06%, and 71.79%; and those of EASI90 were 2.44%, 5.88%, and 23.08% (Figure 1c). There were also no significant differences in them between patients treated with dupilumab and JAK inhibitors.

### 3.3. Serum Galectin-7 Levels in AD Patients Treated with Dupilumab or JAK Inhibitors

**Figure 2:**
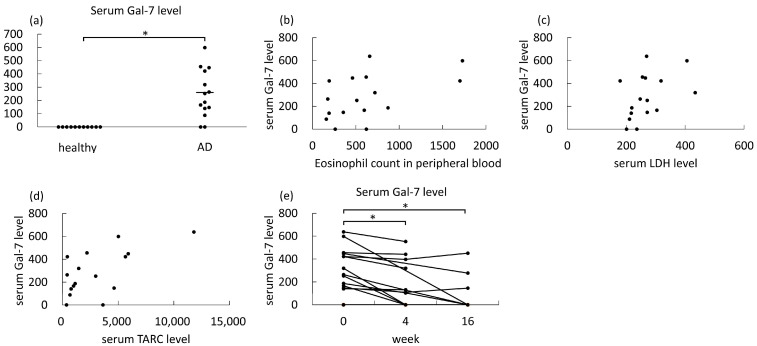
Serum galectin-7 (Gal-7) levels in patients with atopic dermatitis (AD) treated with dupilumab or JAK inhibitors. (**a**) Serum galectin-7 levels in AD patients at baseline (*n* = 16) and healthy controls (*n* = 11). (**b**–**d**) Correlations between serum galectin-7 levels and peripheral blood (**b**), serum lactate dehydrogenase levels (**c**), and serum thymus and activation-regulated chemokine levels (**d**) at baseline (*n* = 16, respectively). (**e**) Serum galectin-7 levels in AD patients at baseline and 4 and 16 weeks after treatment (*n* = 14). The measured values from individual patients are plotted by dots. Bars represent the mean. * *p* < 0.05.

Next, we measured serum galectin-7 levels, which have been reported as a potential biomarker reflecting IL-4/IL-13–induced skin barrier dysfunction and disease severity [15], in patients from whom written informed consent had been obtained. Serum samples were collected from 16 AD patients at baseline and 11 healthy controls (three males and eight females with a mean age of 44.3 ± 16.4 years) from whom written informed consent had also been obtained. Serum galectin-7 levels of AD patients were 284.9 ± 197.2 pg/mL, while serum galectin-7 levels of all healthy controls were below the lower detection limit, and there was a significant difference between them (Figure 2a). Consistent with a previous report [15], serum galectin-7 levels were significantly positively correlated with representative laboratory severity markers of AD, including eosinophil counts in peripheral blood, serum LDH levels, and serum TARC levels (r = 0.47, *p* < 0.05, r = 0.45, *p* < 0.05, and r = 0.54, *p* < 0.01, respectively; Figure 2b–d). Serum samples were obtained from 13 and 8 patients at 4 and 16 weeks after treatment, respectively, and serum galectin-7 levels were significantly reduced after treatment at both time points (Figure 2e).

### 3.4. One-Year Effectiveness of Dupilumab and JAK Inhibitors Based on Patient-Oriented Outcomes

**Figure 3:**
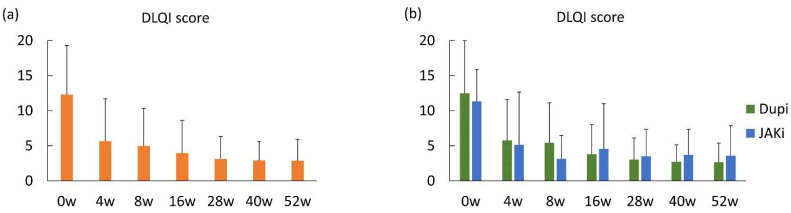
One-year effectiveness of dupilumab and JAK inhibitors based on the Dermatology Life Quality Index (DLQI) score. (**a**) DLQI scores at baseline and 4, 8, 16, 28, 40, and 52 weeks after treatment in the entire population. (**b**) DLQI scores at baseline and 4, 8, 16, 28, 40, and 52 weeks after treatment in patients treated with dupilumab and JAK inhibitors. Data are presented as mean ± standard deviation.

**Figure 4:**
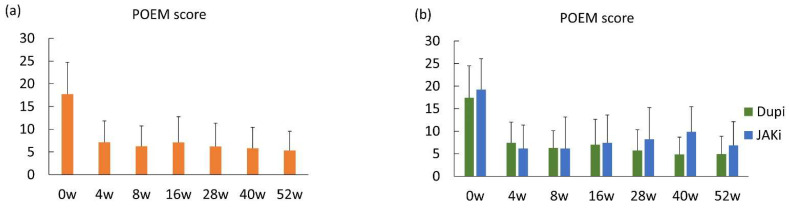
One-year effectiveness of dupilumab and JAK inhibitors based on the patient-oriented eczema measure (POEM) score. (**a**) POEM scores at baseline and 4, 8, 16, 28, 40, and 52 weeks after treatment in the entire population. (**b**) POEM scores at baseline and 4, 8, 16, 28, 40, and 52 weeks after treatment in patients treated with dupilumab and JAK inhibitors. Data are presented as mean ± standard deviation.

Both the DLQI score and POEM score were significantly and rapidly reduced in all patients enrolled at 4 weeks after treatment and gradually decreased until 52 weeks after treatment (Figure 3a and Figure 4a). Mean DLQI scores of five or less, indicating no or small effect on QoL, were achieved at 8, 16, 28, 40, and 52 weeks. Mean POEM scores of seven or less, indicating clear, almost clear, or mild symptoms, were achieved at 8, 28, 40, and 52 weeks. In patients treated with dupilumab, mean DLQI scores of five or less were achieved at 16, 28, 40, and 52 weeks (Figure 3b), while these were achieved at 8, 16, 28, 40, and 52 weeks in patients treated with JAK inhibitors (Figure 3b), suggesting that JAK inhibitors rapidly improved patients’ QoL compared to dupilumab. Mean POEM scores of seven or less were achieved at 8, 16, 28, 40, and 52 weeks in patients treated with dupilumab (Figure 4b). On the other hand, in patients treated with JAK inhibitors, mean POEM scores of seven or less were achieved at 4, 8, and 52 weeks (Figure 4b). These results suggest that the effectiveness of JAK inhibitors on patient-oriented eczema severity may be unstable after 16 weeks of treatment, although rapid effectiveness was observed.

### 3.5. One-Year Stability of Dupilumab and JAK Inhibitors Based on the POEM Score

Given the results of the effectiveness analysis using POEM scores, we counted visits with unstable effectiveness as defined in the Methods section. In patients treated with dupilumab, 71 visits of 27 patients at 28, 40, and 52 weeks after treatment were found, and 8 visits (11.2%) were visits with unstable effectiveness (Table 2). On the other hand, 9 out of 22 visits (40.9%) of 8 patients at 28, 40, and 52 weeks after treatment were visits with unstable effectiveness in patients treated with JAK inhibitors (Table 2). The frequency of visits with unstable effectiveness was higher in patients treated with JAK inhibitors than in those treated with dupilumab.

## 4. Discussion

In a short-term analysis of our cohort, EASI50, EASI75, and EASI90 at 16 weeks were achieved in 87.18%, 71.79%, and 25.64% of patients, respectively, and both dupilumab and JAK inhibitors showed high effectiveness on reduction of the EASI score. Interestingly, there were no significant differences in percentage reductions in the EASI score from baseline between dupilumab and JAK inhibitors in our cohort. To the best of our knowledge, there have been only three real-world studies comparing the effectiveness of dupilumab and JAK inhibitors [14,16,17]. Two of these compared dupilumab with upadacitinib and one compared dupilumab with baricitinib. In our cohort, patients treated with JAK inhibitors included both those treated with upadacitinib and those treated with baricitinib due to the small number of patients. In the multicenter, randomized, double-blinded, clinical trial comparing dupilumab and upadacitnib, EASI75 was achieved in 71.0% of patients treated with upadaticnib and 61.1% of patients treated with dupilumab, and there was a significant difference between them [18]. In one real-world study comparing dupilumab with upadacitinib, EASI75 at 12 weeks in the dupilumab cohort vs. upadacitinib cohort were 51.2% vs. 69.6%, and upadacitinib showed higher effectiveness, similar to a clinical trial [16]. On the other hand, in the other real-world study, EASI75 at 16 weeks was comparable between the two (76.0% in dupilumab cohort vs. 63.6% in upadacitinib cohort) [17]. There have been no clinical trials comparing the efficacy of baricitinib and dupilumab in atopic dermatitis, while based on the meta-analysis of clinical trials, baricitinib seemed to be less effective than dupilumab [19]. However, in the real-world study comparing dupilumab and baricitinib, EASI75 at 16 weeks in the dupilumab cohort vs. baricitinib cohort were 79.2% vs. 71.4%, and no significant difference was found [14]. A combined analysis of patients treated with baricitinib and those treated with upadacitinib may explain the lack of differences in effectiveness at 16 weeks between dupilumab and JAK inhibitors in our cohort. On the other hand, considering the results of some real-world studies, the differences in efficacy between systemic drugs shown or assumed from the results of clinical trials may be masked in real-world practice by concomitant therapies and patient characteristics.

Another characteristic of JAK inhibitors in the treatment of atopic dermatitis is their rapid onset of action, based on the data from clinical trials [7,8,9]. The rapid effectiveness of upadacitinib compared with dupilumab was also demonstrated by EASI score analysis at 4 weeks in two real-world studies comparing dupilumab and upadacitinib [16,17]. Although the percentage reduction and EASI75 at 4 weeks were comparable between dupilumab and JAK inhibitors in our cohort, a mean POEM score of seven or less at 4 weeks was achieved by JAK inhibitors but not by dupilumab, supporting the previously demonstrated rapid action of JAK inhibitors. Similarly, a mean DLQI score of five or less was achieved at 8 weeks by JAK inhibitors, while dupilumab achieved the criteria first at 16 weeks, suggesting that JAK inhibitors can improve the patients’ QoL more rapidly than dupilumab.

We next evaluated the short-term effect of dupilumab and JAK inhibitors on skin barrier dysfunction. In contrast to skin inflammation, there had been limited analysis of the effects of dupilumab on skin barrier dysfunction. Recently, a phase 4 clinical study and several real-world studies have shown that a reduction in transepidermal water loss (TEWL), increase in stratum corneum hydration, and improvement in ceramide synthesis were induced by dupilumab within 16 weeks [20,21,22,23,24,25]. In addition, some studies have revealed increased expression of skin barrier-associated genes, such as filaggrin, loricrin, claudins, and ELOVL3, and decreased expression of type 2 cytokines [26], which impair skin barrier function in lesional skin of AD patients treated with dupilumab [27,28]. However, analysis of other molecules associated with the skin barrier is still limited. The relationship between JAK inhibitors and skin barrier dysfunction also remains to be elucidated, and only a decrease in TEWL by upadacitinib has been reported in a limited number of patients [29]. In this study, we found that dupilumab and JAK inhibitors significantly decreased serum galectin-7 levels in AD patients, which were elevated compared to healthy controls. Galectin-7 was expressed in epidermal keratinocytes in lesional skin of AD and released by IL-4/IL-13 stimulation, resulting in increased serum galectin-7 levels in AD patients [15]. Knockdown of galectin-7 in epidermal keratinocytes induced coarse cell–cell adhesion at the lower epidermis in a 3D–reconstructed epidermis model, and exogenous galectin-7 supplementation did not improve the disrupted adhesion [15]. Thus, endogenous galectin-7 in epidermal keratinocytes contributes to their cell–cell adhesion, and its release by IL-4/IL-13 is one of the causative factors in skin barrier dysfunction in AD. Actually, galectin-7 expression levels in the stratum corneum and serum galectin-7 levels were correlated with TEWL in AD patients [15,30], suggesting that galectin-7 expression is a potential biomarker of IL-4/IL-13–induced skin barrier dysfunction. Collectively, our results may augment previous findings indicating that dupilumab and JAK inhibitors reverse not only skin inflammation but also skin barrier dysfunction in patients with AD.

In addition, we found that serum galectin-7 levels were correlated with AD severity markers, such as serum LDH levels, serum TARC levels, and eosinophil counts in peripheral blood in this study. Similarly, Umayahara et al. reported that serum galectin-7 levels were correlated with serum LDH levels, serum TARC levels, and circulating eosinophil percentages in a previous report [15]. Thus, serum galectin-7 levels may not only be a marker of skin barrier dysfunction but also a marker of disease severity in AD.

We finally assessed the long-term stability of effectiveness up to one year based on the POEM score. The sustained long-term efficacy of dupilumab and JAK inhibitors was confirmed in clinical trials [31,32,33,34]. In real-world settings, some studies have suggested a slight lack of persistence with JAK inhibitors. Hagino et al. evaluated the real-world effectiveness of upadacitinib in patients with moderate-to-severe AD up to 96 weeks and detected a slight reduction in effectiveness at time points later than 36 weeks in a specific population, that is, in systemic therapy-experienced patients receiving upadacitinib at a dose of 15 mg daily [35]. In a real-world study comparing dupilumab with upadacitinib by Yang et al. [17], EASI75 at 24 and 40 weeks were higher in dupilumab-treated patients than those in patients treated with 15 mg/day upadacitinib, although EASI75 at 8 and 16 weeks were comparable, suggesting a relative lack of persistence of upadacitinib. On the other hand, the long-term sustained effectiveness of JAK inhibitors in the real-world has also been reported [36,37], and a definite conclusion has not been made yet. In this study, we found that the frequency of visits with unstable effectiveness, defined as a visit with a three-point or greater increase in the POEM score compared to the previous visit at 28, 40, and 52 weeks, was higher in patients treated with JAK inhibitors than those with dupilumab. Therefore, the effectiveness of JAK inhibitors may be unstable compared to dupilumab. JAK inhibitors may be more susceptible to factors associated with patient characteristics, such as renal function, hepatic function, and drug interactions, and blood levels may be more unstable compared to dupilumab given the nature of the drugs. This may be the reason for the instability of JAK inhibitors. However, in our study, the POEM score decreased significantly at the next visit of a visit with unstable effectiveness in some patients without a change in treatment, suggesting that instability of efficacy does not necessarily mean loss of efficacy, and that continuation may be considered.

This study had limitations. First, this was a single-institution retrospective study with several potential biases. Second, the number of patients included in this study was not sufficiently large. Further investigations in a large-scale multicenter study should be conducted to make a definite conclusion. However, to the best of our knowledge, this is the fourth real-world study to compare the characteristics of effectiveness of dupilumab and JAK inhibitors for moderate-to-severe atopic dermatitis.

## 5. Conclusions

Both dupilumab and JAK inhibitors showed high effectiveness on skin inflammation and decreased a marker of skin barrier dysfunction within 16 weeks in our cohort. JAK inhibitors improved patient-reported outcomes more quickly than dupilumab, but instability of effectiveness during 16 and 52 weeks was higher with JAK inhibitors.

## Figures and Tables

**Table 1:** Baseline demographics and clinical characteristics of patients with atopic dermatitis who were treated with dupilumab and Janus kinase (JAK) inhibitors.

	All Patients (*n* = 55)	Patients Treated with Dupilumab (*n* = 45)	Patients Treated with JAK Inhibitors (*n* = 10)
Age (years)	32.2 ± 15.6	32.7 ± 15.2	29.7 ± 17.9
Sex (male: female)	41: 14	35: 10	6: 4
IGA score	3.13 ± 0.70	3.16 ± 0.71	3 ± 0.67
EASI score	25.6 ± 7.03	25.78 ± 6.47	24.78 ± 9.39
%BSA (%)	55.29 ± 14.40	55.47 ± 15.33	54.50 ± 9.69
POEM score	17.74 ± 6.99	17.41 ± 7.06	19.22 ± 6.83
DLQI score	12.28 ± 7.03	12.49 ± 7.49	11.33 ± 4.56
Atopic comorbidities	47 (85.4%)	37 (82.2%)	10 (100%)

Continuous variables were presented as mean ± standard deviation. Abbreviations: BSA, body surface area; DLQI, Dermatology Life Quality Index; EASI, eczema area and severity index; IGA, Investigator’s Global Assessment; POEM, patient-oriented eczema measurement.

**Table 2:** The number of visits with or without unstable effectiveness at 28, 40, and 52 weeks in patients treated with dupilumab and Janus kinase (JAK) inhibitors.

	Visits with Unstable Effectiveness	Visits Without Unstable Effectiveness
patients treated with dupilumab	8	63
patients treated with JAK inhibitors	9	13

## Data Availability

The original contributions presented in this study are included in the article. Further inquiries can be directed to the corresponding author.
